# Porcine Circovirus 3 Detection in Aborted Fetuses and Stillborn Piglets from Swine Reproductive Failure Cases

**DOI:** 10.3390/v13020264

**Published:** 2021-02-09

**Authors:** Viviane Saporiti, Laura Valls, Jaime Maldonado, Mónica Perez, Florencia Correa-Fiz, Joaquim Segalés, Marina Sibila

**Affiliations:** 1 IRTA, Centre de Recerca en Sanitat Animal (CReSA, IRTA-UAB), Campus de la Universitat Autònoma de Barcelona, 08193 Bellaterra, Spain; viviane.saporiti@irta.cat (V.S.); monica.perez@irta.cat (M.P.); flor.correa@irta.cat (F.C.-F.); marina.sibila@irta.cat (M.S.); 2 OIE Collaborating Centre for the Research and Control of Emerging and Re-emerging Swine Diseases in Europe (IRTA-CReSA), Bellaterra, 08193 Barcelona, Spain; 3 Laboratorios Hipra, S.A., Amer, 17170 Girona, Spain; laura.valls@hipra.com (L.V.); jaime.maldonado@hipra.com (J.M.); 4 Departament de Sanitat i Anatomia Animals, Universitat Autònoma de Barcelona (UAB), Bellaterra, 08193 Barcelona, Spain; 5 UAB, Centre de Recerca en Sanitat Animal (CReSA, IRTA-UAB), Campus de la Universitat Autònoma de Barcelona, 08193 Bellaterra, Spain

**Keywords:** porcine circovirus 3 (PCV-3), reproductive failure, aborted fetuses, stillborn, quantitative PCR, in situ hybridization, histopathology

## Abstract

Porcine circovirus 3 (PCV-3) has been widely detected in healthy and diseased pigs; among different pathologic conditions, the strongest evidence of association comes from reproductive disease cases. However, simple viral detection does not imply the causality of the clinical conditions. Detection of PCV-3 within lesions may provide stronger evidence of causality. Thus, this study aimed to assess the frequency of PCV-3 detection in tissues from fetuses/stillborn piglets in cases of reproductive problems in domestic swine, as well as the histopathologic assessment of fetal tissues. Fetuses or stillborn piglets from 53 cases of reproductive failure were collected and analyzed by PCV-3 qPCR. The presence of porcine reproductive and respiratory syndrome virus (PRRSV), porcine circovirus 2 (PCV-2), and porcine parvovirus 1 (PPV1) was also checked. PCV-3 qPCR positive samples with a high viral load were tested by PCV-3 in situ hybridization (ISH), sequenced, and phylogenetically analyzed. PCV-3 DNA was detected in 18/53 (33.9%) reproductive failure cases and in 16 of them PCV-3 was the only pathogen found. PCV-2 DNA was found in 5/53 (9.4%), PRRSV RNA in 4/53 (7.5%) and PPV1 was not detected. Four out of the six PCV-3 qPCR-positive cases with Ct value <30 were positive when tested by ISH. In these samples, PCV-3 was detected within mild histopathologic lesions, such as arteritis and periarteritis in multiple tissues. The present work emphasizes the need to include PCV-3 as a potential causative agent of reproductive failure in swine.

## 1. Introduction

Reproductive failure in sows represents an important drawback for the pig production sector, causing great economical losses worldwide [1,2,3]. When reproductive diseases affect dams during late-term gestation, the failure is manifested as abortions, stillborn and/or weak-born piglets or premature farrowing. A number of different viruses are associated with reproductive problems, such as the porcine reproductive and respiratory syndrome virus (PRRSV), porcine parvovirus 1 (PPV1), porcine circovirus 2 (PCV-2), Aujeszky’s disease virus (ADV), influenza virus A (IAV), encephalomyocarditis virus (EMCV), and porcine enteroviruses (PEV), among others [2]. Lately, porcine circovirus 3 (PCV-3) has also been proposed as a pathogen associated with reproductive disease causation [4,5,6,7,8,9,10,11,12].

PCV-3 was first described in 2015 and, since then, its role in different diseases has been debated. Although the viral DNA has been found in healthy pigs [13,14] as well as in animals displaying various clinical conditions [15,16,17,18], some recent reports have shown PCV-3 is most frequently associated with reproductive problems [4,5,6,7,8,9,10,11,12]. However, the mere detection of an enzootic virus in animals with clinical conditions is not sufficient to demonstrate its disease causality [4]. Despite the worldwide ubiquitous distribution of PCV-3 [13,19], there is little evidence of PCV-3 detection within lesions of diseased animals [4,20]. In consequence, it is of great importance to determine the frequency of the virus in farms with reproductive problems and assess its potential causality in these problems.

Thus, the present study aimed to investigate the frequency of PCV-3 detection in fetal/stillborn tissues of reproductive cases from Spanish farms. In addition, histopathologic description of positive cases and in situ detection of the virus was performed.

## 2. Materials and Methods

### 2.1. Sample Selection

Tissue samples from 53 cases of reproductive failure submitted to the Veterinary Diagnostic Laboratory (DIAGNOS) of HIPRA in Spain (Amer, Spain) (*n* = 51), and to the *Servei de Diagnòstic de Patologia Veterinària de la Facultat de Veterinària de la Universitat Autònoma de Barcelona* (UAB, Bellaterra, Spain) (*n* = 2) between 2019 and 2020 were included in this study. The number of aborted/stillborn fetuses investigated per case ranged from 1 to 13. Cases originated from different conventional farms located in 15 different Spanish provinces.

From each fetus/stillborn piglet, fresh (heart, lung, spleen, kidney, thymus, and liver) and fixed tissues (heart, lung, spleen, kidney, thymus, liver, intestine, and in some cases cerebrum and cerebellum) were collected during the necropsy. Fresh tissues were pooled (per case) and homogenized (10% *w*/*v* in PBS), while the formalin-fixed tissues were handled for histopathologic evaluation. The crown-rump length (CRL) of the specimens (aborted fetus or stillborn) was measured to estimate the pregnancy timing.

### 2.2. DNA Extraction, PCV-2, and PCV-3 qPCR

DNA extraction was done from 200 μL of supernatant from the pooled macerated tissues (heart, lung, spleen, kidney, thymus, and liver) using MagMAx™ Pathogen RNA/DNA Kit (Applied Biosystems^®^, Foster City, CA, USA) following the manufacturer’s protocol.

A real-time quantitative PCR (qPCR) analysis was performed targeting PCV-3 as previously described [21,22]. The qPCR results were expressed in log10 of PCV-3 DNA copies/µL of supernatant of macerated tissue samples; the limit of detection of the technique is one copy of DNA/μL [21,22].

PCV-2 qPCR was performed using LSI VetMAX™ Porcine PCV2 Quant Kit (Applied Biosystems^®^, Foster City, CA, USA), according to the manufacturer’s protocol. The qPCR results were expressed in log10 of PCV-2 DNA copies/mL of supernatant of macerated tissue samples; the limit of detection of the technique is 4 log10 copies of DNA/mL as indicated by the manufacturer.

### 2.3. PRRSV and PPV1 Detection

RNA extraction was done from 100 μL of supernatant from the same pooled macerated tissues as described before using the RNeasy Mini kit (Qiagen, Hilden, Germany) following the manufacturer’s instructions. The PPRSV real-time PCR analysis was performed using a previously described technique [23].

Two hundred microliters of the same pooled macerated tissues were used to perform DNA extraction by using the QIAamp DNA Mini Kit (Qiagen, Hilden, Germany) following the manufacturer’s protocol. PPV1 presence was assessed using real-time PCR analysis adapted from [24].

### 2.4. Histopathology, PCV-2 Immunohistochemistry, and PCV-3 In Situ Hybridization

Available tissues from each case (heart, lung, spleen, kidney, thymus, liver, intestine, cerebrum, and/or cerebellum) were fixed in 10% neutral buffered formalin. Fixed samples were dehydrated, embedded in paraffin wax, sectioned at 4 µm, and stained with hematoxylin and eosin (HE). Subsequent paraffin block cuts were used to assess PCV-2 and PCV-3 by immunohistochemistry (IHC) and in situ hybridization (ISH), respectively, on selected samples.

PCV-2 qPCR positive samples were tested by PCV-2 IHC [25].

PCV-3 qPCR positive samples with Ct ≤ 30 were analyzed by a PCV-3 ISH using the RNAscope^®^ 2.5 HD Reagent Kit-RED (Advanced Cell Diagnostics, Newark, CA, USA) according to the manufacturer’s instructions [26]. Paraffin-embedded tissues from the selected cases were mounted on Dako Flex^®^ slides (Ref. K8020). The slide sections were dried overnight in an incubator at 37 °C. Slides were deparaffinized by being immersed two times for 5 min in xylene followed by two times for 3 min in 100% alcohol and then air dried for 10 min at RT. The pre-treatment was made with RNAscope^®^ Hydrogen Peroxide at RT for 10 min, rinsed with distilled water, and then boiled for 20 min at 99 °C in the RNAscope^®^ 1X Target Retrieval Reagent. Subsequently, slides were rinsed in distilled water, and washed in fresh 100% alcohol for 1 min. After air drying, a hydrophobic barrier was drawn around each tissue section using the ImmEdge™ pen. Afterwards, the RNAscope^®^ Protease Plus was applied and incubated for 10 min at 40 °C in the HybEZ™ Oven, and washed with distilled water. The PCV-3 Rep target probe (catalogue No. 491021) as well as the negative DapB probe (catalogue No. 310043) were pre-heated for 10 min at 40 °C and then hybridized for 2 h at 40 °C in the HybEZ™ Oven. Slides were washed with RNAscope^®^ 1X Wash Buffer and followed by six amplification steps (RNAscope ^®^ 2.5 AMP 1–6) interspersed with washes of RNAscope^®^ 1X Wash Buffer. Slides were incubated with red chromogenic detection solution for 10 min at RT, followed by a counterstain with 50% hematoxylin, and rinsed with tap water for 10 min. After air drying, slides were quickly mounted by submerging them into fresh pure xylene and using EcoMount (Biocare Medical, Pacheco, CA, USA) to coverslip them. All tested slides (one or two) per case were also assayed with a negative control probe, as recommended by the manufacturer’s protocols. A positive control with the PCV-3 Rep probe was also used for each ISH batch performed. The amount of cells stained by the ISH was scored semi quantitatively with ”–“ for no infected cells, “+” for low amount (<10 labelled cells as a mean of 5 fields examined at x200 magnification), “++” for medium amount (10–50 labelled cells), and “+++” for a high amount of cells (>50 labelled cells) per tissue sample containing the PCV-3 genome.

### 2.5. PCV-3 and PCV-2 Sequencing and PCV-3 Phylogenetic Analysis

All qPCR PCV-2 positive samples were selected for sequencing the ORF2 to establish the potential genotype involved using primers described by Oliver-Ferrando et al. (2016) [27], with slight modifications [22].

Positive PCV-3 qPCR samples that showed a Ct ≤ 30 were selected for complete genome sequencing. PCV-3 DNA was amplified using a previously described panel of primers [28], purified with ExoSAP-IT^®^ Express PCR product Cleanup Kit (Applied Biosystems^®^, Foster City, CA, USA) according to the manufacturer’s protocol, and sequenced by the Sanger method (ABI3730XL-Macrogen Europe, Madrid, Spain). The quality of the sequences was analyzed by Finch TV software and trimmed in BioEdit v. 7.2.6 [29]. To achieve the PCV-3 complete genome, the amplicons were assembled using the online version of MAFFT version 7 [30] and aligned with a set of published reference samples selected by Franzo et al. (2020) [31] (available at: https://www.mdpi.com/1999-4915/12/3/265/s1, accessed on 15 October 2020) using ClustalW available in BioEdit vs. 7.2.6 [29]. PCV-3 ORF2 gene and the translated ORF2 amino acid (aa) region were also aligned with the same selected references. For the phylogenetic analysis, the best substitution method was chosen based on the lowest Bayesian Information Criterion (BIC) score calculated using MEGA X software [32], either for the complete genome analysis as for the ORF2 or the ORF2 aa analysis. The maximum likelihood phylogenetic tree of the complete genome was constructed using the Tamura–Nei model plus Gamma and I (G+I) distribution, while the ORF2 analysis was performed using Hasegawa–Kishino–Yano (HKY) model [33] plus Gamma distribution, and the translated ORF2 region was analyzed using Jones–Taylor–Thornton’s model [34] plus Gamma distribution. All trees were built with 1000 bootstrap replicates using MEGAX software [32]. The nucleotide (nt) and aa identity matrices among sequences were obtained using Clustal Omega [35]. The PCV-3 sequences obtained in this study are available at the NCBI GenBank with the accession numbers MW167063–MW167068.

## 3. Results

### 3.1. Virus Detection

From the 53 collected cases of reproductive failure, 18 (33.9%) were PCV-3 qPCR positive. From these 18 samples, 8 showed quantifiable viral loads (from 0.3 to 5.7 log10 copies/μL of supernatant of macerated tissue samples). Among these 8 samples, 6 had high viral loads with Ct levels ≤30 (from 2.9 to 5.7 log10 copies/μL).

PCV-2 DNA was detected in 5 out of the 53 cases (9.4%). These 5 positive cases had low viral loads, below the quantification limit of the technique (BLQ). PRRSV RNA was found in 4 out of the 53 cases (7.5%), and PPV1 was not detected. Only two out of the 53 (3.8%) cases displayed coinfection (Table 1): one case was positive for PCV-3 and PRRSV, and the other one to PCV-3, PCV-2, and PRRSV (Supplementary Table S1).

The CRL of the fetus/stillborn piglets ranged from 6 to 32 cm. Most of the fetal death occurred in the last third of gestation.

### 3.2. Histologic Evaluation, PCV-2 IHC, and PCV-3 ISH

In most of the cases, autolysis and no histologic lesions were observed. Few cases (6 out of 53) had histologic lesions; three animals had mild multifocal interstitial pneumonia (case Nos. 5, 22, and 52) and three had systemic lymphoplasmacytic periarteritis (case Nos. 3, 40, and 52).

Four out of five PCV-2 qPCR positive samples did not show histological lesions (only case No. 22 showed interstitial pneumonia) and were negative by PCV-2 IHC. No histological lesions were observed in the four PRRSV qPCR positive cases.

PCV-3 was detected by ISH in 4 out of the 6 selected samples with high viral loads (PCV-3 qPCR Ct ≤ 30) (Table 2). These four PCV-3 ISH positive cases (case Nos. 3, 35, 40, and 52) had no evidence of coinfections. Two cases, Nos. 36 and 39, with qPCR Ct < 30 but negative by ISH, had marked signs of autolysis in tissues.

None of the studied viruses was found in one case with interstitial pneumonia (case No. 5).

**Figure 1:**
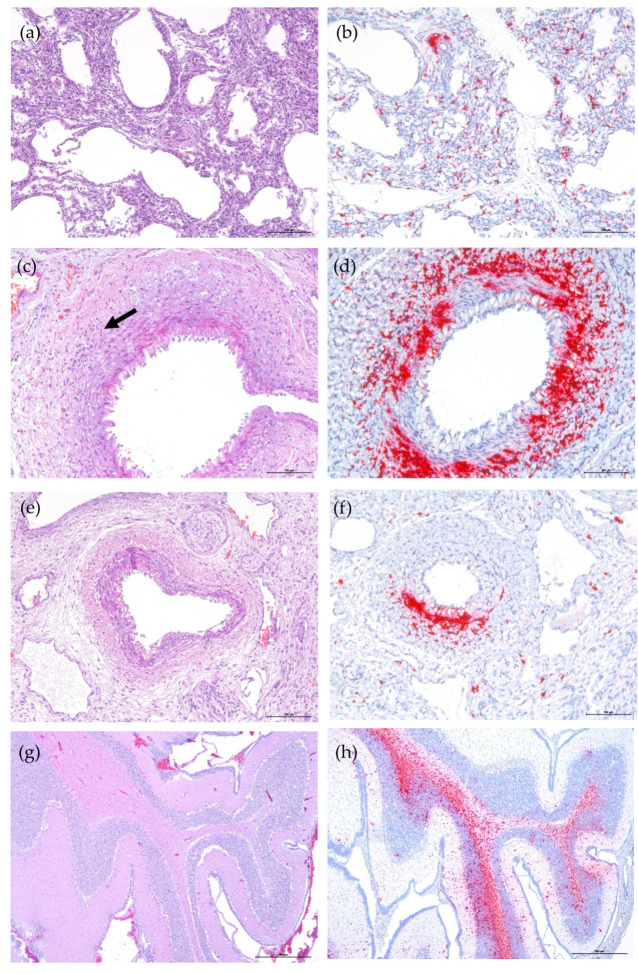
Histology (H&E stain, **a**,**c**,**e**,**g**) and PCV-3 ISH (hematoxylin counterstain, **b**,**d**,**f**,**h**) results. (**a**) Fetal lung from case No. 3 with no apparent histopathologic lesions. (**b**) PCV-3 genome in macrophage-like cells and in a perivascular area in lung fetus from case No. 3. (**c**) Moderate tunica media swelling in a splenic artery with a mild lymphocytic infiltration at perivascular and arteriolar locations (arrow) of a stillborn piglet from case No. 52. (**d**) PCV-3 detection in smooth muscle of spleen artery and perivascular inflammation in the same fetus. (**e**) Kidney–pelvis artery of a stillborn piglet from case No. 52. (**f**) PCV-3 detection in smooth muscle and in lamina propria of a kidney artery of the same fetus. (**g**) Cerebellum from a stillborn piglet with no apparent histopathologic lesions of fetus from case No. 52. (**h**) High amount of PCV-3 nucleic acid in cerebellum white matter and mild-to-moderate in grey matter of the same animal.

The PCV-3 genome was mainly found in the smooth muscle cells of arteries in different tissues, and also in macrophage-like cells in lung and kidney (Figure 1). Fetuses from three out of the four ISH-positive cases showed mild inflammation (mainly mild lymphocytic infiltration (LI)) in the same area of PCV-3 ISH labeling (Nos. 3, 40, and 52). The most abundant labeled cells were found in case Nos. 40 and 52. Case No. 40 showed almost all the analyzed tissues positive to PCV-3 by ISH; however, only the heart tissue showed evident histopathologic lesions. In case No. 52, PCV-3 was detected in all tested tissues, and arteritis and perivascular inflammation were observed in most of them (Figure 1c–f), except in cerebrum and cerebellum (Figure 1g,h). Tissues such as the heart and spleen of case No. 3 and the lung of case No. 35 contained low amounts of labeled cells without any histological lesions.

### 3.3. Sequencing and Phylogenetic Analysis

None of the PCV-2 positive samples were successfully sequenced due the low amount of virus in the investigated samples; therefore, PCV-2 genotyping was unable to be assessed.

In the six samples with high PCV-3 viral loads (Ct ≤ 30), sequencing was successfully performed. Thus, the nucleotide identity of the PCV-3 complete genome of the analyzed samples (*n* = 6) demonstrated that five out the six showed high identity between each other (Table 3). However, one of the sequences (case No. 40) showed lower identity (96.7% to 97.8%) when compared to the other five obtained herein and also when compared to the set of reference sequences proposed by Franzo et al. (2020) (Table 3). Thus, this sequence slightly exceeded the maximum within-genotype genetic distance of 3% at the complete genome analysis in order to be classified as PCV-3a, as previously proposed [31] (Franzo et al., 2020).

**Figure 2:**
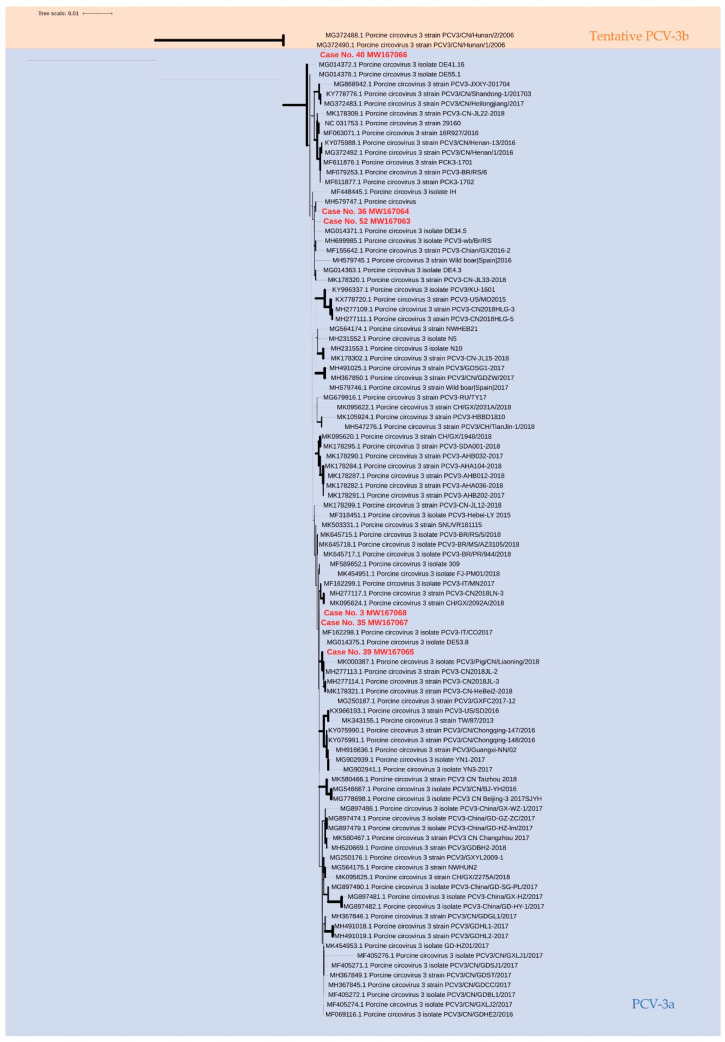
Phylogenetic analysis of PCV-3 complete genome sequences from this study, and the PCV-3 reference sequences from Franzo et al. [31]. The maximum likelihood tree was constructed with the Tamura–Nei model with Gamma plus I distribution (1000 replicates). The width of the branches is proportional to bootstrap values, and the scale bar indicates nucleotide substitutions per site. Sequences obtained in the present study are colored in red; sequences classified as PCV-3a are in the blue shadow, and the sequences of a tentative PCV-3b in orange shadow.

The phylogenetic analysis of the whole PCV-3 genome displayed two main clusters (Figure 2). Five out of the six sequences analyzed in this study belonged to the same cluster, together with the reference sequences previously genotyped as PCV-3a [31]. The sample from case No. 40 showed the highest number of single nucleotide polymorphisms (SNP) found throughout the complete genome (Supplementary Table S2) when compared to the other cases and located in a separated branch in the phylogenetic tree (Figure 2). Similar results were found when ORF2 sequences were phylogenetically analyzed, as case No. 40 also clustered with samples classified as PCV-3a but in a separated branch of the phylogenetic tree (Figure S1). When the number of SNPs was compared throughout the ORF regions, ORF2 was the one containing the highest number of SNPs (9–26 SNPs in the *cap* gene, while there were 1–8 SNPs in the *rep* gene).

Moreover, the *cap* region was translated and the aa sequences from the inferred ORF2 protein were phylogenetically analyzed (Figure S2). All of them were grouped in the same cluster including case 40, in agreement with the low number of SNPs (26 SNPs) found that corresponded to five non-synonymous mutations in this sequence (Supplementary Table S2).

## 4. Discussion

In the present study on reproductive failure cases, PCV-3 was the viral agent detected at a higher frequency, whereas PPV1 was not found, and PCV-2 and PRRSV were detected in less than 10% of these cases. Notably, the global rate of infection by viruses of the present study is fairly high (around 45%). Although it cannot be proven that the pathogens found herein were the cause of the reproductive losses in the studied farms, the results found in the present work fit well with the fact that an average of more than 50% of reproductive failure cases are not of infectious origin [3]. There are also a significant number of bacterial pathogens that may account for reproductive disorders [2]. The three viruses studied here, apart from PCV-3, are already well recognized as putative causative agents of reproductive disease [36,37,38,39], and the results obtained in the present studies reinforce [4,20,40] the potential of PCV-3 as a pathogen able to cause reproductive problems, as suggested elsewhere [4,20,40]. The present study did not aim at establishing potential bacteria causing reproductive disorders; however, no lesions compatible with bacterial infections were observed during histologic evaluation in the studied tissues of fetuses and stillborn piglets.

From all tested cases, only five were positive to PCV-2 by qPCR. These PCV-2 positive cases had very low viral load in tissues, did not present any typical pathologic lesion associated to this infection, such as myocarditis [41], and were negative by IHC. Altogether, results suggest that the reproductive problems detected in these cases were probably not associated with this virus. Since PRRSV was the third pathogen detected most frequently in the samples assessed, it cannot be ruled out that this virus was involved in the reproductive problems observed in affected farms. However, no evidence of significant histologic lesions was observed in tissues from fetuses infected by PRRSV. A similar study performed in 2005, in which the presence of different viruses (PRRSV, ADV, PPV1 and PCV-2) in reproductive failures was investigated, concluded that PRRSV was the main pathogen responsible for late-term abortion in Spain as this virus was detected in 9 out of 100 reproductive failures [42]. Therefore, the results obtained in both Spanish studies demonstrate that the frequency of PRRSV detection in reproductive failure cases in Spanish herds is similar fifteen years later. Maldonado et al. (2005) also found a low frequency of PCV-2 DNA (1/100) by PCR and no positive cases by ISH. The lack of PPV1 detection in the studied cases also coincides with the abovementioned study and could be explained by the wide use of vaccination against this agent [42]. However, the comparison of PCV-3 frequency with the study of Maldonado et al. (2005) is not possible due the fact that this virus was not described at that time, even though it is known through retrospective studies that PCV-3 has been circulating in Europe since the mid-1990s at least [13]. Thus, if PCV-3 would have been investigated at that time, we hypothesize that it would probably have been found.

PCV-3 DNA has been widely detected in samples from healthy animals [10,13,14,43,44] as well as in mummified fetuses and stillborn piglets from farms without reproductive problems [19]. The simple assessment of viral DNA in cases of reproductive problems also has been widely performed [5,6,7,9,10], but the detection of a viral genome alone does not imply the causality of a disease. Therefore, the next step was to investigate the presence of PCV-3 within lesions in fetuses/stillborn piglets in farms displaying reproductive problems, as suggested [4]. Two out of the six cases with high loads of PCV-3 in tissues showed a lack of histological lesions. These results are not surprising since other studies have already mentioned the lack of lesions even in the presence of great amounts of PCV-3 DNA [7,40]. A Hungarian group found high loads of PCV-3 in tissues from aborted and weak-born piglets with no gross lesions [6]. Indeed, this lack of gross lesions in aborted fetuses is not uncommon, thus microscopic lesions are a more valuable indicator of viral abortion [2,4]. Additionally, Faccini et al. (2017), also found high PCV-3 loads (10^7^ genome copies/µL) in pools of lung tissues from fetuses and stillborn piglets, without detection of microscopic lesions. However, in the present study, fetuses from PCV-3 ISH positive cases (Nos. 3, 35, 40, and 52) had mild-to-moderate inflammation (mainly lymphoplasmacytic infiltration) in arteries in different tissues (case Nos. 3, 35, and 52), or mild myocarditis and endocarditis (case No. 40). These findings suggest a PCV-3 tropism mainly for vasculature, similar to what has been described in piglets with multisystemic inflammation [4,11,20,40]. Some of the animals from these latest reports also showed lesions compatible with porcine dermatitis nephropathy syndrome (PDNS) [4,11,12]. Moreover, PCV-3 detection through ISH was also observed in a wasting pig in South Korea showing respiratory distress with perivascular and peribronchiolar lymphocytic infiltration [8]. This animal was also positive to other pathogens such as PRRSV, *Streptoccocus suis* and *Mycoplasma hyopneumoniae* [8]. Additionally a recent experimental PCV-3 inoculation performed in the USA provided evidences that PCV-3 is able to produce multisystemic inflammation as well as subclinical infections [40]. Specifically, all animals of two different inoculated groups had prolonged viremia, but only 2/4 and 3/4 showed histopathologic lesions [40]. Thus, Temeeyasen et al. [40] suggested that the PCV-3 pathogenesis is complex and of multifactorial nature. The results presented herein, where most of the cases (4 out of 6) with high PCV-3 loads had histopathologic findings, support the abovementioned statement that the presence and also the severity of the lesions might be multifactorial [4,20,40]. Moreover, Temeeyasen et al. [40] speculated that the higher number of positive tissues by qPCR compared to positive ISH could be explained by viremia and was not due to in situ viral replication.

Franzo et al. [31] have proposed a single PCV-3 genotype (PCV-3a) after analyzing most of the PCV-3 available sequences in public databases. Three characteristics were suggested for the definition of PCV-3 genotypes: (1) the limit of the maximum within-genotype raw genetic distance of 3% at complete genome analysis, (2) 6% at the ORF2 level, and (3) a 90% minimum of bootstrap. Thus, the phylogenetic analysis of the six PCV-3 sequences obtained in this study demonstrated that five out of the six sequences were unequivocally classified as PCV-3a [31]. All five sequences showed high nt identity between each other as well as when compared to the set of selected samples classified as PCV-3a by Franzo et al. [31]. The five sequences also clustered together in all constructed phylogenetic trees. However, case No. 40 showed the nt identity of the complete genome sequence close to the limit of the genetic distance proposed to define the PCV-3 genotype [31]. In fact, this sequence only strictly fulfilled one out of the three characteristics (the second one mentioned above) to classify PCV-3 sequences within the unique proposed genotype. Therefore, the sequence of case No. 40 was within the limit of the newest proposed genotype. This finding reinforces the importance of the continuous surveillance of the sequences circulating in the field.

The present work demonstrated the presence of the PCV-3 genome within (mild-to-moderate) histological lesions of aborted fetuses. Thus, PCV-3 should be considered as a potential causative pathogen for reproductive failure. Based on the present findings, further studies are needed to elucidate the specific pathogenesis of PCV-3 infection of the pregnant sow, the frequency at which the virus can cause lesions and disease, as well as its economic impact on the sector.

## Figures and Tables

**Table 1:** Frequency and range of fetal/stillborn piglet size of qPCR positive cases by pathogen and coinfections.

Agent	Positive (Out of 53)	%	CRL (cm) Range
PCV-3	16	30.2	15–32
PCV-2	4	7.5	16–30
PRRSV	2	3.8	20–28
PCV-3/PRRSV	1	1.9	23–27
PCV-3/PCV-2/PRRSV	1	1.9	6–22 *

* The highly variable range was due to the presence of mummified fetuses among the aborted ones.

**Table 2:** Crown-to-rump (CRL) range of the 6 PCV-3 qPCR positive samples with high viral loads by case no., results of PCV-3 tests (qPCR and ISH), and histologic lesions.

Case No.	CRL (cm) Range	PCV-3 Ct Values (log10 Copies/μL)	PCV-3 ISH Results		Histological Lesions
3	22	24.4 (3.5)	Heart	+	Heart: No lesions
			Lung	++	Lung: mild LI in arteries
			Intestine	+	Intestine: mild perivascular LI
			Spleen	+	Spleen: No lesions
			Kidney	+	Kidney: mild LI in pelvis
			Liver	–	Liver: No lesions
35	15–20	24.3 (4.7)	Heart	–	Heart: No lesions
			Lung	+	Lung: tunica media swelling in lung arteries’ wall
			Kidney	–	Kidney: No lesions
			Liver	–	Liver: No lesions
36	6–22	24.3 (3.5)	Heart	–	Heart: Autolysis
			Kidney	–	Kidney: Autolysis
			Liver	–	Liver: Autolysis
39	15–25	26.5 (2.9)	Heart	–	Heart: Autolysis
			Lung	–	Lung: Autolysis
			Intestine	–	Intestine: Autolysis
			Spleen	–	Spleen: Autolysis
			Kidney	–	Kidney: Autolysis
			Liver	–	Liver: Autolysis
			Umbilical cord	–	Umbilical cord: Autolysis
40	16–23	18.0 (5.5)	Heart	+++	Heart: mild LI in smooth muscles of arteries, mild lymphoplasmacytic endocarditis and myocarditis
			Lung	++	Lung: No lesions
			Intestine	+	Intestine: No lesions
			Kidney	+	Kidney: No lesions
			Liver	++	Liver: Autolysis
			Thymus	–	Thymus: No lesions
52	27–28	16.9 (5.71)	Heart	++	Heart: mild LI in perivascular connective tissue
			Lung	++	Lung: hemorrhage, IP’ and mild LI in arteries
			Spleen	++	Spleen: hemorrhage, mild LI’ and vacuolization in smooth muscles of arteries’ wall
			Kidney	++	Kidney: mild LI in smooth muscles of arteries
			Liver	++	Liver: mild LI in arteries and hepatocytes with vacuolization
			Cerebrum	+++	Neuropil vacuolization
			Cerebellum	++	Multifocal cortical hemorrhages

–: non-infected cell; +: low amount of PCV-3 positive cells; ++: medium amount of PCV-3 positive cells; +++: high amount of PCV-3 positive cells. LI: lymphocytic infiltration; IP: interstitial pneumonia.

**Table 3:** Range in percentage (%) of complete genome of nt, ORF2 nt, and ORF2 aa identity between PCV-3 sequences retrieved in this study and selected reference sequences [31] (Franzo et al., 2020).

Sequences from Case No.	Sequences from Case Nos. 3, 35, 36, 39, 52 (%)			Set of Published Reference Samples Selected by Franzo et al. (2020) (%)		
	Complete Genome	ORF2	ORF2 aa	Complete Genome	ORF2	ORF2 aa
**3, 35, 36, 39, 52**	99.2–100	98.9–100	98.6–100	98.4–100	97.2–100	95.3–100
**40**	97.5–97.7	95.9–96.1	96.7–97.2	96.7–97.8	95.4–96.6	94.9–98.1

## Data Availability

Full data are provided in the supplementary materials.

## Supplementary Materials

The following are available online at https://www.mdpi.com/1999-4915/13/2/264/s1, Figure S1: Phylogenetic analysis of PCV-3 ORF2 nt sequences from this study, and the PCV-3 reference sequences from Franzo et al. [31]. The maximum likelihood tree was constructed with the Hasegawa–Kishino–Yano model with Gamma distribution (lowest BIC score) at 1000 replicates. The width of the branches is proportional to the bootstrap values, and the scale bar indicates nucleotide substitutions per site. The six sequences obtained herein are colored in red; sequences classified as PCV-3a are in the blue shadow and the sequences of a tentative PCV-3b in orange shadow. Figure S2: PCV-3 phylogenetic analysis tree of aa of CAP protein from sequences from this study, and the PCV-3 references sequences from Franzo et al. [31]. The maximum likelihood tree was constructed with Jones–Taylor–Thornton’s model with Gamma distribution (lowest BIC score) at 1000 replicates. The width of the branches is proportional to bootstrap values, and the scale bar indicates nucleotide substitutions per site. The six sequences obtained herein are colored in red; sequences classified as PCV-3a are in the blue shadow and the sequences of a tentative PCV-3b in orange shadow. Table S1: Number of fetuses or stillborn piglets, range of crown-to-rump length (CRL), and RT-qPCR or qPCR results obtained in those cases positive at least for one virus. Table S2: Table of SNPs of sequences retrieved in this study compared to the PCV-3 reference of NCBI (NC031753). The genomic positions are displayed in 5′-3′ except for the non-synonymous SNPs from the *cap* gene (displayed 3′-5’).

## References

[1] Nathues H., Alarcon P., Rushton J., Jolie R., Fiebig K., Jimenez M., Geurts V., Nathues C. (2017). Cost of porcine reproductive and respiratory syndrome virus at individual farm level—An economic disease model. Prev. Vet. Med..

[2] Althouse G.C., Kauffold J., Rossow S., Zimmerman J.J., Karriker L.A., Ramirez A., Schwartz K.J., Stevenson G.W., Zhang. J. (2019). Diseases of the reproductive system. Diseases of Swine.

[3] Christianson W.T. (1992). Stillbirths, mummies, abortions, and early embryonic death. Vet. Clin. N. Am. Food Anim. Pract..

[4] Arruda B., Piñeyro P., Derscheid R., Hause B., Byers E., Dion K., Long D., Sievers C., Tangen J., Williams T. (2019). PCV3-associated disease in the United States swine herd. Emerg. Microbes Infect..

[5] Dal Santo A.C., Cezario K.C., Bennemann P.E., Machado S.A., Martins M. (2020). Full-genome sequences of porcine circovirus 3 (PCV3) and high prevalence in mummified fetuses from commercial farms in Brazil. Microb. Pathog..

[6] Deim Z., Dencso L., Erdélyi I., Valappil S.K., Varga C., Pósa A., Makrai L., Rákhely G. (2019). Porcine circovirus type 3 detection in a Hungarian pig farm experiencing reproductive failures. Vet. Rec..

[7] Faccini S., Barbieri I., Gilioli A., Sala G., Gibelli L.R., Moreno A., Sacchi C., Rosignoli C., Franzini G., Nigrelli A. (2017). Detection and genetic characterization of Porcine circovirus type 3 in Italy. Transbound. Emerg. Dis..

[8] Kim S.H., Park J.Y., Jung J.Y., Kim H.Y., Park Y.R., Lee K.K., Lyoo Y.S., Yeo S.G., Park C.K. (2018). Detection and genetic characterization of porcine circovirus 3 from aborted fetuses and pigs with respiratory disease in Korea. J. Vet. Sci..

[9] Tochetto C., Lima D.A., Varela A.P.M., Loiko M.R., Paim W.P., Scheffer C.M., Herpich J.I., Cerva C., Schmitd C., Cibulski S.P. (2018). Full-genome sequence of porcine circovirus type 3 recovered from serum of sows with stillbirths in Brazil. Transbound. Emerg. Dis..

[10] Zou Y., Zhang N., Zhang J., Zhang S., Jiang Y., Wang D., Tan Q., Yang Y., Wang N. (2018). Molecular detection and sequence analysis of porcine circovirus type 3 in sow sera from farms with prolonged histories of reproductive problems in Hunan, China. Arch. Virol..

[11] Phan T.G., Giannitti F., Rossow S., Marthaler D., Knutson T., Li L., Deng X., Resende T., Vannucci F., Delwart E. (2016). Detection of a novel circovirus PCV3 in pigs with cardiac and multi-systemic inflammation. Virol. J..

[12] Palinski R., Piñeyro P., Shang P., Yuan F., Guo R., Fang Y., Byers E., Hause B.M. (2017). A novel porcine circovirus distantly related to known circoviruses is associated with porcine dermatitis and nephropathy syndrome and reproductive failure. J. Virol..

[13] Klaumann F., Franzo G., Sohrmann M., Correa-Fiz F., Drigo M., Núñez J.I., Sibila M., Segalés J. (2018). Retrospective detection of porcine circovirus 3 (PCV-3) in pig serum samples from Spain. Transbound. Emerg. Dis..

[14] Stadejek T., Woźniak A., Miłek D., Biernacka K. (2017). First detection of porcine circovirus type 3 on commercial pig farms in Poland. Transbound. Emerg. Dis..

[15] Kedkovid R., Woonwong Y., Arunorat J., Sirisereewan C., Sangpratum N., Lumyai M., Kesdangsakonwut S., Teankum K., Jittimanee S., Thanawongnuwech R. (2018). Porcine circovirus type 3 (PCV3) infection in grower pigs from a Thai farm suffering from porcine respiratory disease complex (PRDC). Vet. Microbiol..

[16] Qi S., Su M., Guo D., Li C., Wei S., Feng L., Sun D. (2019). Molecular detection and phylogenetic analysis of porcine circovirus type 3 in 21 provinces of China during 2015–2017. Transbound. Emerg. Dis..

[17] Shen H., Liu X., Zhang P., Wang L., Liu Y., Zhang L., Liang P., Song C. (2018). Genome characterization of a porcine circovirus type 3 in south China. Transbound. Emerg. Dis..

[18] Zhai S.L., Zhou X., Zhang H., Hause B.M., Lin T., Liu R., Chen Q.L., Wei W.K., Lv D.H., Wen X.H. (2017). Comparative epidemiology of porcine circovirus type 3 in pigs with different clinical presentations. Virol. J..

[19] Saporiti V., Martorell S., Cruz T.F., Klaumann F., Correa-Fiz F., Balasch M., Sibila M., Segalés J. (2020). Frequency of detection and phylogenetic analysis of porcine circovirus 3 (Pcv-3) in healthy primiparous and multiparous sows and their mummified fetuses and stillborn. Pathogens.

[20] Mora-Díaz J., Piñeyro P., Shen H., Schwartz K., Vannucci F., Li G., Arruda B., Giménez-Lirola L. (2020). Isolation of PCV3 from perinatal and reproductive cases of PCV3-associated disease and in vivo characterization of PCV3 replication in CD/CD growing Pigs. Viruses.

[21] Franzo G., Legnardi M., Centelleghe C., Tucciarone C.M., Cecchinato M., Cortey M., Segalés J., Drigo M. (2018). Development and validation of direct PCR and quantitative PCR assays for the rapid, sensitive, and economical detection of porcine circovirus 3. J. Vet. Diagnostic Investig..

[22] Saporiti V., Cruz T.F., Correa-Fiz F., Núñez J.I., Sibila M., Segalés J. (2019). Similar frequency of porcine circovirus 3 (PCV-3) detection in serum samples of pigs affected by digestive or respiratory disorders and age-matched clinically healthy pigs. Transbound. Emerg. Dis..

[23] Martínez E., Riera P., Sitjà M., Fang Y., Oliveira S., Maldonado J. (2008). Simultaneous detection and genotyping of porcine reproductive and respiratory syndrome virus (PRRSV) by real-time RT-PCR and amplicon melting curve analysis using SYBR Green. Res. Vet. Sci..

[24] Miao L., Zhang C.-F., Chen C.-M., Cui S.-J. (2009). Real-time PCR to detect and analyze virulent PPV loads in artificially challenged sows and their fetuses. Vet. Microbiol..

[25] Grau-Roma L., Hjulsager C.K., Sibila M., Kristensen C.S., López-Soria S., Enøe C., Casal J., Bøtner A., Nofrarías M., Bille-Hansen V. (2009). Infection, excretion and seroconversion dynamics of porcine circovirus type 2 (PCV2) in pigs from post-weaning multisystemic wasting syndrome (PMWS) affected farms in Spain and Denmark. Vet. Microbiol..

[26] Wang F., Flanagan J., Su N., Wang L.C., Bui S., Nielson A., Wu X., Vo H.T., Ma X.J., Luo Y. (2012). RNAscope: A novel in situ RNA analysis platform for formalin-fixed, paraffin-embedded tissues. J. Mol. Diagnostics.

[27] Oliver-Ferrando S., Segalés J., López-Soria S., Callén A., Merdy O., Joisel F., Sibila M. (2016). Evaluation of natural porcine circovirus type 2 (PCV2) subclinical infection and seroconversion dynamics in piglets vaccinated at different ages. Vet. Res..

[28] Fux R., Söckler C., Link E.K., Renken C., Krejci R., Sutter G., Ritzmann M., Eddicks M. (2018). Full genome characterization of porcine circovirus type 3 isolates reveals the existence of two distinct groups of virus strains. Virol. J..

[29] Hall T.A. (1999). Bioedit. Nucleic Acids Symp. Ser..

[30] Katoh K., Rozewicki J., Yamada K.D. (2019). MAFFT online service: Multiple sequence alignment, interactive sequence choice and visualization. Brief. Bioinform..

[31] Franzo G., Delwart E., Fux R., Hause B., Su S., Zhou J.Y., Segalés J. (2020). Genotyping porcine circovirus 3 (PCV-3) nowadays: Does it make sense?. Viruses.

[32] Kumar S., Stecher G., Li M., Knyaz C., Tamura K. (2018). MEGA X: Molecular evolutionary genetics analysis across computing platforms. Mol. Biol. Evol..

[33] Hasegawa M., Kishino H., Yano T. (1985). AKI dating of the human-ape splitting by a molecular clock of mitochondrial DNA. J. Mol. Evol..

[34] Jones D.T., Taylor W.R., Thornton J.M. (1992). The rapid generation of mutation data matrices. Comput. Appl. Biosci..

[35] Sievers F., Higgins D.G. (2014). Multiple sequence alignment methods. Bioinformatics.

[36] Brunborg I.M., Jonassen C.M., Moldal T., Bratberg B., Lium B., Koenen F., Schönheit J. (2007). Association of myocarditis with high viral load of porcine circovirus type 2 in several tissues in cases of fetal death and high mortality in piglets. A case study. J. Vet. Diagn. Investig..

[37] Madson D.M., Patterson A.R., Ramamoorthy S., Pal N., Meng X.J., Opriessnig T. (2009). Effect of porcine circovirus type 2 (PCV2) vaccination of the dam on PCV2 replication in utero. Clin. Vaccine Immunol..

[38] Hermann J.R., Muñoz-Zanzi C.A., Roof M.B., Burkhart K., Zimmerman J.J. (2005). Probability of porcine reproductive and respiratory syndrome (PRRS) virus infection as a function of exposure route and dose. Veter Microbiol..

[39] Truyen U., Streck A.F., Truyen U., Streck A.F. (2019). Parvoviruses. Diseases of Swine.

[40] Temeeyasen G., Lierman S., Arruda B.L., Main R., Vannucci F., Gimenez L.G. (2020). Pathogenicity and immune response against porcine circovirus type 3 infection in caesarean—deprived pigs. J. Gen. Virol..

[41] Segalés J. (2012). Porcine circovirus type 2 (PCV2) infections: Clinical signs, pathology and laboratory diagnosis. Virus Res..

[42] Maldonado J., Segalés J., Martínez-Puig D., Calsamiglia M., Riera P., Domingo M., Artigas C. (2005). Identification of viral pathogens in aborted fetuses and stillborn piglets from cases of swine reproductive failure in Spain. Vet. J..

[43] Franzo G., Legnardi M., Hjulsager C.K., Klaumann F., Larsen L.E., Segales J., Drigo M. (2018). Full-genome sequencing of porcine circovirus 3 field strains from Denmark, Italy and Spain demonstrates a high within-Europe genetic heterogeneity. Transbound. Emerg. Dis..

[44] Ye X., Berg M., Fossum C., Wallgren P., Blomström A.L. (2018). Detection and genetic characterisation of porcine circovirus 3 from pigs in Sweden. Virus Genes.

