# Supplementary material for: Porcine Circovirus 3 Detection in Aborted Fetuses and Stillborn Piglets from Swine Reproductive Failure Cases
Source: Viruses. 2021 Feb 9;13(2):264. doi: 10.3390/v13020264 (PMC7915229; doi:10.3390/v13020264)
Supplement: Supplementary file 1 [file viruses-13-00264-s001.pdf]

Supplementary Table S1. Number of fetuses or stillborn piglets, range of crown-to-rump length (CRL) and RT-qPCR or qPCR results obtained in those cases positive at least for one virus.

Case No.	Number of fetuses	CRL (cm) range	PCV-3 Ct values (log10 copies/μL)	PCV-2 Ct values (log10 copies/mL)	PRRSV Ct values
2	5	22-30	Neg	37.0 (BLQ)	Neg
3	1	22	24.4 (3.5)	Neg	Neg
4	2	22-28	35.6 (BLQ)	Neg	Neg
6	1	23	Neg	Neg	32.9
9	1	30	37.7 (BLQ)	Neg	Neg
13	3	23	37.5 (BLQ)	Neg	Neg
14	13	20-28	Neg	Neg	24.91
17	8	26-32	37.4 (BLQ)	Neg	Neg
22	2	16-21	Neg	35.5 (BLQ)	Neg
24	5	23	38.2 (BLQ)	Neg	Neg
30	2	22-25	Neg	38.4 (BLQ)	Neg
32	6	25-28	37.4 (BLQ)	Neg	Neg
35	5	15-20	24.3 (4.7)	Neg	Neg
36	4	6-22	24.3 (3.5)	37.2 (BLQ)	32.7
39	11	15-25	26.5 (2.9)	Neg	Neg
40	3	16-23	18.0 (5.5)	Neg	Neg
41	2	22-25	Neg	38.3 (BLQ)	Neg
42	10	23-27	33.8 (0.6)	Neg	27.1
43	2	22	35.5 (BLQ)	Neg	Neg
44	10	17-23	36.5 (BLQ)	Neg	Neg
47	6	23-27	34.7 (0.30)	Neg	Neg
48	2	23	37.9 (BLQ)	Neg	Neg
49	2	15	37.8 (BLQ)	Neg	Neg
52	6	27-28	16.9 (5.71)	Neg	Neg

BLQ: Below the quantification limit

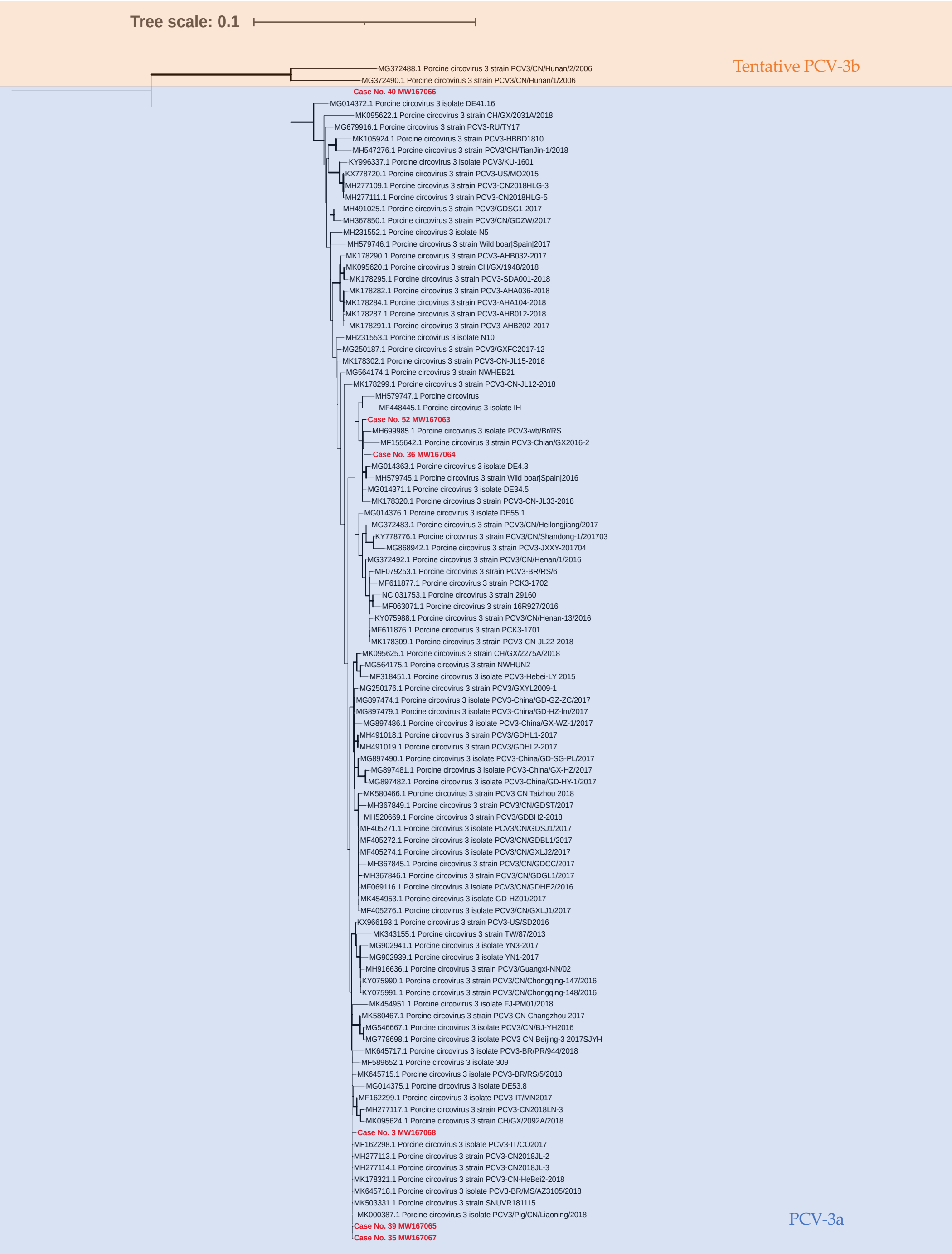
Tree scale: 0.1
Tentative PCV-3b
MG372488.1 Porcine circovirus 3 strain PCV3/CN/Hunan/2/2006
MG372490.1 Porcine circovirus 3 strain PCV3/CN/Hunan/1/2006
Case No. 40 MW167066
MG014372.1 Porcine circovirus 3 isolate DE41.16
MK095622.1 Porcine circovirus 3 strain CH/GX/2031A/2018
MG679916.1 Porcine circovirus 3 strain PCV3-RU/TY17
MK105924.1 Porcine circovirus 3 strain PCV3-HBBD1810
MH547276.1 Porcine circovirus 3 strain PCV3/CH/TianJin-1/2018
KY996337.1 Porcine circovirus 3 isolate PCV3/KU-1601
KX778720.1 Porcine circovirus 3 strain PCV3-US/MO2015
MH277109.1 Porcine circovirus 3 strain PCV3-CN2018HLG-3
MH277111.1 Porcine circovirus 3 strain PCV3-CN2018HLG-5
MH491025.1 Porcine circovirus 3 strain PCV3/GDSG1-2017
MH367850.1 Porcine circovirus 3 strain PCV3/CN/GDZW/2017
MH231552.1 Porcine circovirus 3 isolate N5
MH579746.1 Porcine circovirus 3 strain Wild boar|Spain|2017
MK178290.1 Porcine circovirus 3 strain PCV3-AHB032-2017
MK095620.1 Porcine circovirus 3 strain CH/GX/1948/2018
MK178295.1 Porcine circovirus 3 strain PCV3-SDA001-2018
MK178282.1 Porcine circovirus 3 strain PCV3-AHA036-2018
MK178284.1 Porcine circovirus 3 strain PCV3-AHA104-2018
MK178287.1 Porcine circovirus 3 strain PCV3-AHB012-2018
MK178291.1 Porcine circovirus 3 strain PCV3-AHB202-2017
MH231553.1 Porcine circovirus 3 isolate N10
MG250187.1 Porcine circovirus 3 strain PCV3/GXFC2017-12
MK178302.1 Porcine circovirus 3 strain PCV3-CN-JL15-2018
MG564174.1 Porcine circovirus 3 strain NWHEB21
MK178299.1 Porcine circovirus 3 strain PCV3-CN-JL12-2018
MH579747.1 Porcine circovirus
MF448445.1 Porcine circovirus 3 isolate IH
Case No. 52 MW167063
MH699985.1 Porcine circovirus 3 isolate PCV3-wb/Br/RS
MF155642.1 Porcine circovirus 3 strain PCV3-Chian/GX2016-2
Case No. 36 MW167064
MG014363.1 Porcine circovirus 3 isolate DE4.3
MH579745.1 Porcine circovirus 3 strain Wild boar|Spain|2016
MG014371.1 Porcine circovirus 3 isolate DE34.5
MK178320.1 Porcine circovirus 3 strain PCV3-CN-JL33-2018
MG014376.1 Porcine circovirus 3 isolate DE55.1
MG372483.1 Porcine circovirus 3 strain PCV3/CN/Heilongjiang/2017
KY778776.1 Porcine circovirus 3 strain PCV3/CN/Shandong-1/201703
MG868942.1 Porcine circovirus 3 strain PCV3-JXXY-201704
MG372492.1 Porcine circovirus 3 strain PCV3/CN/Henan/1/2016
MF079253.1 Porcine circovirus 3 strain PCV3-BR/RS/6
MF611877.1 Porcine circovirus 3 strain PCK3-1702
NC 031753.1 Porcine circovirus 3 strain 29160
MF063071.1 Porcine circovirus 3 strain 16R927/2016
KY075988.1 Porcine circovirus 3 strain PCV3/CN/Henan-13/2016
MF611876.1 Porcine circovirus 3 strain PCK3-1701
MK178309.1 Porcine circovirus 3 strain PCV3-CN-JL22-2018
MK095625.1 Porcine circovirus 3 strain CH/GX/2275A/2018
MG564175.1 Porcine circovirus 3 strain NWHUN2
MF318451.1 Porcine circovirus 3 isolate PCV3-Hebei-LY 2015
MG250176.1 Porcine circovirus 3 strain PCV3/GXYL2009-1
MG897474.1 Porcine circovirus 3 isolate PCV3-China/GD-GZ-ZC/2017
MG897479.1 Porcine circovirus 3 isolate PCV3-China/GD-HZ-lm/2017
MG897486.1 Porcine circovirus 3 isolate PCV3-China/GX-WZ-1/2017
MH491018.1 Porcine circovirus 3 strain PCV3/GDHL1-2017
MH491019.1 Porcine circovirus 3 strain PCV3/GDHL2-2017
MG897490.1 Porcine circovirus 3 isolate PCV3-China/GD-SG-PL/2017
MG897481.1 Porcine circovirus 3 isolate PCV3-China/GX-HZ/2017
MG897482.1 Porcine circovirus 3 isolate PCV3-China/GD-HY-1/2017
MK580466.1 Porcine circovirus 3 strain PCV3 CN Taizhou 2018
MH367849.1 Porcine circovirus 3 strain PCV3/CN/GDST/2017
MH520669.1 Porcine circovirus 3 strain PCV3/GDBH2-2018
MF405271.1 Porcine circovirus 3 isolate PCV3/CN/GDSJ1/2017
MF405272.1 Porcine circovirus 3 isolate PCV3/CN/GDBL1/2017
MF405274.1 Porcine circovirus 3 isolate PCV3/CN/GXLJ2/2017
MH367845.1 Porcine circovirus 3 strain PCV3/CN/GDCC/2017
MH367846.1 Porcine circovirus 3 strain PCV3/CN/GDGL1/2017
MF069116.1 Porcine circovirus 3 isolate PCV3/CN/GDHE2/2016
MK454953.1 Porcine circovirus 3 isolate GD-HZ01/2017
MF405276.1 Porcine circovirus 3 isolate PCV3/CN/GXLJ1/2017
KX966193.1 Porcine circovirus 3 strain PCV3-US/SD2016
MK343155.1 Porcine circovirus 3 strain TW/87/2013
MG902941.1 Porcine circovirus 3 isolate YN3-2017
MG902939.1 Porcine circovirus 3 isolate YN1-2017
MH916636.1 Porcine circovirus 3 strain PCV3/Guangxi-NN/02
KY075990.1 Porcine circovirus 3 strain PCV3/CN/Chongqing-147/2016
KY075991.1 Porcine circovirus 3 strain PCV3/CN/Chongqing-148/2016
MK454951.1 Porcine circovirus 3 isolate FJ-PM01/2018
MK580467.1 Porcine circovirus 3 strain PCV3 CN Changzhou 2017
MG546667.1 Porcine circovirus 3 isolate PCV3/CN/BJ-YH2016
MG778698.1 Porcine circovirus 3 isolate PCV3 CN Beijing-3 2017SJYH
MK645717.1 Porcine circovirus 3 isolate PCV3-BR/PR/944/2018
MF589652.1 Porcine circovirus 3 isolate 309
MK645715.1 Porcine circovirus 3 isolate PCV3-BR/RS/5/2018
MG014375.1 Porcine circovirus 3 isolate DE53.8
MF162299.1 Porcine circovirus 3 isolate PCV3-IT/MN2017
MH277117.1 Porcine circovirus 3 strain PCV3-CN2018LN-3
MK095624.1 Porcine circovirus 3 strain CH/GX/2092A/2018
Case No. 3 MW167068
MF162298.1 Porcine circovirus 3 isolate PCV3-IT/CO2017
MH277113.1 Porcine circovirus 3 strain PCV3-CN2018JL-2
MH277114.1 Porcine circovirus 3 strain PCV3-CN2018JL-3
MK178321.1 Porcine circovirus 3 strain PCV3-CN-HeBei2-2018
MK645718.1 Porcine circovirus 3 isolate PCV3-BR/MS/AZ3105/2018
MK503331.1 Porcine circovirus 3 strain SNUVR181115
MK000387.1 Porcine circovirus 3 isolate PCV3/Pig/CN/Liaoning/2018
Case No. 39 MW167065
Case No. 35 MW167067
PCV-3a

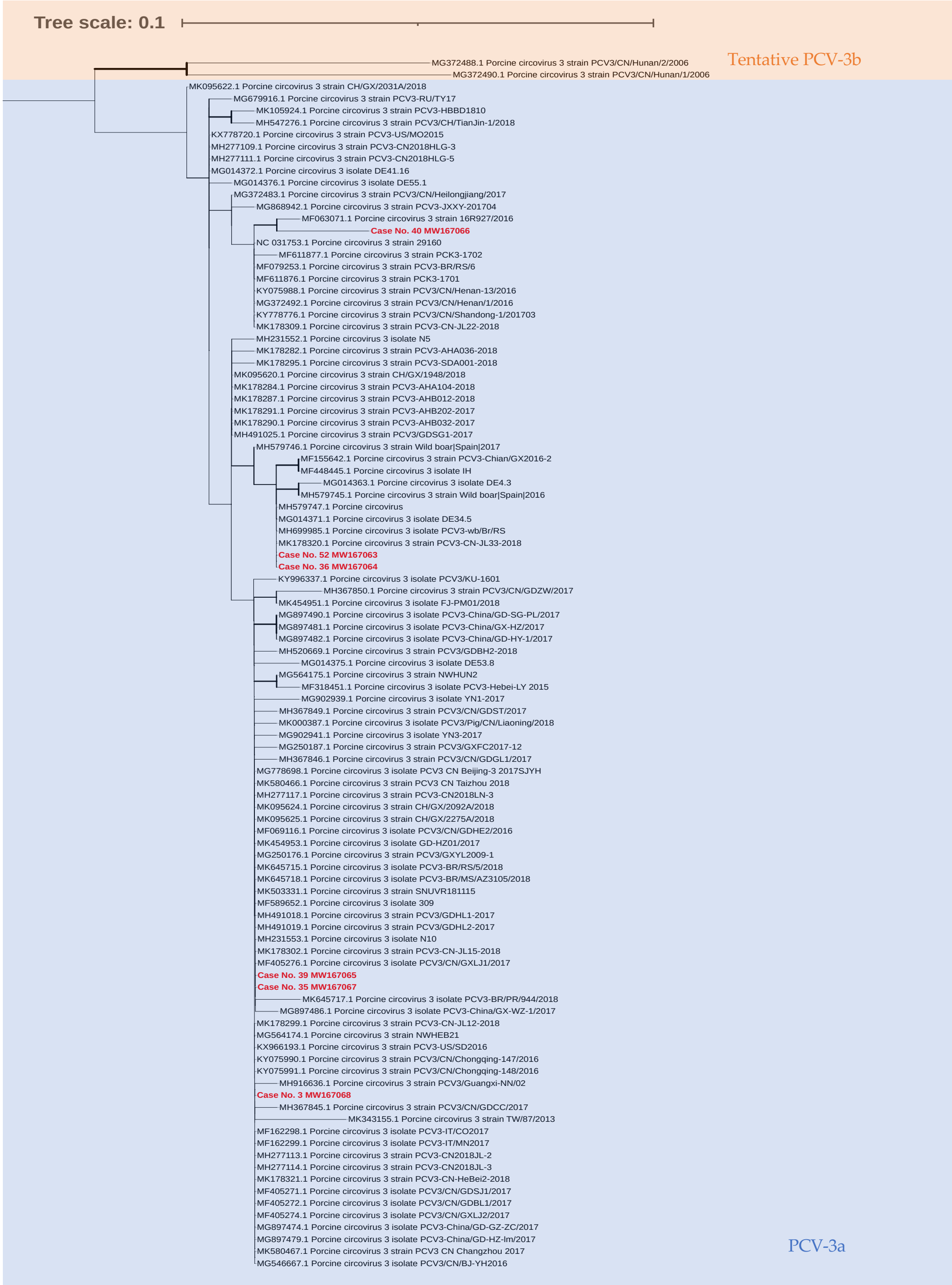

Tree scale: 0.1
Tentative PCV-3b
MG372488.1 Porcine circovirus 3 strain PCV3/CN/Hunan/2/2006
MG372490.1 Porcine circovirus 3 strain PCV3/CN/Hunan/1/2006
MK095622.1 Porcine circovirus 3 strain CH/GX/2031A/2018
MG679916.1 Porcine circovirus 3 strain PCV3-RU/TY17
MK105924.1 Porcine circovirus 3 strain PCV3-HBBD1810
MH547276.1 Porcine circovirus 3 strain PCV3/CH/TianJin-1/2018
KX778720.1 Porcine circovirus 3 strain PCV3-US/MO2015
MH277109.1 Porcine circovirus 3 strain PCV3-CN2018HLG-3
MH277111.1 Porcine circovirus 3 strain PCV3-CN2018HLG-5
MG014372.1 Porcine circovirus 3 isolate DE41.16
MG014376.1 Porcine circovirus 3 isolate DE55.1
MG372483.1 Porcine circovirus 3 strain PCV3/CN/Heilongjiang/2017
MG868942.1 Porcine circovirus 3 strain PCV3-JXXY-201704
MF063071.1 Porcine circovirus 3 strain 16R927/2016
Case No. 40 MW167066
NC 031753.1 Porcine circovirus 3 strain 29160
MF611877.1 Porcine circovirus 3 strain PCK3-1702
MF079253.1 Porcine circovirus 3 strain PCV3-BR/RS/6
MF611876.1 Porcine circovirus 3 strain PCK3-1701
KY075988.1 Porcine circovirus 3 strain PCV3/CN/Henan-13/2016
MG372492.1 Porcine circovirus 3 strain PCV3/CN/Henan/1/2016
KY778776.1 Porcine circovirus 3 strain PCV3/CN/Shandong-1/201703
MK178309.1 Porcine circovirus 3 strain PCV3-CN-JL22-2018
MH231552.1 Porcine circovirus 3 isolate N5
MK178282.1 Porcine circovirus 3 strain PCV3-AHA036-2018
MK178295.1 Porcine circovirus 3 strain PCV3-SDA001-2018
MK095620.1 Porcine circovirus 3 strain CH/GX/1948/2018
MK178284.1 Porcine circovirus 3 strain PCV3-AHA104-2018
MK178287.1 Porcine circovirus 3 strain PCV3-AHB012-2018
MK178291.1 Porcine circovirus 3 strain PCV3-AHB202-2017
MK178290.1 Porcine circovirus 3 strain PCV3-AHB032-2017
MH491025.1 Porcine circovirus 3 strain PCV3/GDSG1-2017
MH579746.1 Porcine circovirus 3 strain Wild boar|Spain|2017
MF155642.1 Porcine circovirus 3 strain PCV3-Chian/GX2016-2
MF448445.1 Porcine circovirus 3 isolate IH
MG014363.1 Porcine circovirus 3 isolate DE4.3
MH579745.1 Porcine circovirus 3 strain Wild boar|Spain|2016
MH579747.1 Porcine circovirus
MG014371.1 Porcine circovirus 3 isolate DE34.5
MH699985.1 Porcine circovirus 3 isolate PCV3-wb/Br/RS
MK178320.1 Porcine circovirus 3 strain PCV3-CN-JL33-2018
Case No. 52 MW167063
Case No. 36 MW167064
KY996337.1 Porcine circovirus 3 isolate PCV3/KU-1601
MH367850.1 Porcine circovirus 3 strain PCV3/CN/GDZW/2017
MK454951.1 Porcine circovirus 3 isolate FJ-PM01/2018
MG897490.1 Porcine circovirus 3 isolate PCV3-China/GD-SG-PL/2017
MG897481.1 Porcine circovirus 3 isolate PCV3-China/GX-HZ/2017
MG897482.1 Porcine circovirus 3 isolate PCV3-China/GD-HY-1/2017
MH520669.1 Porcine circovirus 3 strain PCV3/GDBH2-2018
MG014375.1 Porcine circovirus 3 isolate DE53.8
MG564175.1 Porcine circovirus 3 strain NWHUN2
MF318451.1 Porcine circovirus 3 isolate PCV3-Hebei-LY 2015
MG902939.1 Porcine circovirus 3 isolate YN1-2017
MH367849.1 Porcine circovirus 3 strain PCV3/CN/GDST/2017
MK000387.1 Porcine circovirus 3 isolate PCV3/Pig/CN/Liaoning/2018
MG902941.1 Porcine circovirus 3 isolate YN3-2017
MG250187.1 Porcine circovirus 3 strain PCV3/GXFC2017-12
MH367846.1 Porcine circovirus 3 strain PCV3/CN/GDGL1/2017
MG778698.1 Porcine circovirus 3 isolate PCV3 CN Beijing-3 2017SJYH
MK580466.1 Porcine circovirus 3 strain PCV3 CN Taizhou 2018
MH277117.1 Porcine circovirus 3 strain PCV3-CN2018LN-3
MK095624.1 Porcine circovirus 3 strain CH/GX/2092A/2018
MK095625.1 Porcine circovirus 3 strain CH/GX/2275A/2018
MF069116.1 Porcine circovirus 3 isolate PCV3/CN/GDHE2/2016
MK454953.1 Porcine circovirus 3 isolate GD-HZ01/2017
MG250176.1 Porcine circovirus 3 strain PCV3/GXYL2009-1
MK645715.1 Porcine circovirus 3 isolate PCV3-BR/RS/5/2018
MK645718.1 Porcine circovirus 3 isolate PCV3-BR/MS/AZ3105/2018
MK503331.1 Porcine circovirus 3 strain SNUVR181115
MF589652.1 Porcine circovirus 3 isolate 309
MH491018.1 Porcine circovirus 3 strain PCV3/GDHL1-2017
MH491019.1 Porcine circovirus 3 strain PCV3/GDHL2-2017
MH231553.1 Porcine circovirus 3 isolate N10
MK178302.1 Porcine circovirus 3 strain PCV3-CN-JL15-2018
MF405276.1 Porcine circovirus 3 isolate PCV3/CN/GXLJ1/2017
Case No. 39 MW167065
Case No. 35 MW167067
MK645717.1 Porcine circovirus 3 isolate PCV3-BR/PR/944/2018
MG897486.1 Porcine circovirus 3 isolate PCV3-China/GX-WZ-1/2017
MK178299.1 Porcine circovirus 3 strain PCV3-CN-JL12-2018
MG564174.1 Porcine circovirus 3 strain NWHEB21
KX966193.1 Porcine circovirus 3 strain PCV3-US/SD2016
KY075990.1 Porcine circovirus 3 strain PCV3/CN/Chongqing-147/2016
KY075991.1 Porcine circovirus 3 strain PCV3/CN/Chongqing-148/2016
MH916636.1 Porcine circovirus 3 strain PCV3/Guangxi-NN/02
Case No. 3 MW167068
MH367845.1 Porcine circovirus 3 strain PCV3/CN/GDCC/2017
MK343155.1 Porcine circovirus 3 strain TW/87/2013
MF162298.1 Porcine circovirus 3 isolate PCV3-IT/CO2017
MF162299.1 Porcine circovirus 3 isolate PCV3-IT/MN2017
MH277113.1 Porcine circovirus 3 strain PCV3-CN2018JL-2
MH277114.1 Porcine circovirus 3 strain PCV3-CN2018JL-3
MK178321.1 Porcine circovirus 3 strain PCV3-CN-HeBei2-2018
MF405271.1 Porcine circovirus 3 isolate PCV3/CN/GDSJ1/2017
MF405272.1 Porcine circovirus 3 isolate PCV3/CN/GDBL1/2017
MF405274.1 Porcine circovirus 3 isolate PCV3/CN/GXLJ2/2017
MG897474.1 Porcine circovirus 3 isolate PCV3-China/GD-GZ-ZC/2017
MG897479.1 Porcine circovirus 3 isolate PCV3-China/GD-HZ-Im/2017
MK580467.1 Porcine circovirus 3 strain PCV3 CN Changzhou 2017
MG546667.1 Porcine circovirus 3 isolate PCV3/CN/BJ-YH2016
PCV-3a

Region	UTR							REP													UTR									
Genomic position (NC031753)	36	40	54	76	79	115	178	317	515	557	579	662	725	800	837	876	890	968	1007	1061	1136	1148	1149	1160	1174	1182	1183	1328	1329	1333
Nt	G	G	A	C	T	G	T	A	C	A	T	G	A	T	T	C	C	T	A	G	G	G	T	A	G	G	C	A	T	C
Case 3											G		T		C							T		T						
											S122A																			
Case 35											G		T		C									T						
											S122A																			
Case 36															C								A	T			A			
Case 39							C			T	G	T	T		C									T						T
											S122A																			
Case 40		A	T	T	A	T		G	A					A	C	T		A	C	T	T			T	A	A	G	C	C	
Case 52	C				A										C		T							T						

Region	CAP																																			
Genomic position (NC031753)	1345	1384	1405	1426	1438	1482	1483	1519	1533	1579	1583	1585	1600	1670	1693	1696	1714	1726	1732	1751	1756	1758	1795	1819	1825	1834	1846	1900	1901	1902	1910	1933	1939	1941	1951	1968
Nt	G	G	C	C	T	A	G	A	G	A	T	A	C	A	C	A	T	T	T	G	T	C	G	A	T	T	T	T	C	G	G	T	C	T	T	C
Case 3									T			C						C		C	C					A	G		T	T	A					
									L150I											T77S									R27K		A24V					
Case 35									T									C		C	C					A	G		T	T	A					
									L150I											T77S									R27K		A24V					
Case 36									T				T	T				C		C			T			A	G			T		G				
									L150I					F104Y						T77S																
Case 39									T									C		C	C					A	G		T	T	A					
									L150I											T77S									R27K		A24V					
Case 40	T	A	T	T	C	T	A	T		T	C					T	G	C	A	C		A		C	C	A	G	C		T			T	G	C	G
						S167T					K133R									T77S		A75S														A5P
Case 52									T					T	T			C		C						A	G			T		G				
									L150I					F104Y						T77S																
